# Report on the 7th scientific meeting of the Association for the Advancement of Young Academics in Neurology (NEUROWIND e.V.) held in Motzen, Germany, October 30–November 1, 2015

**DOI:** 10.1186/s13231-016-0017-y

**Published:** 2016-03-16

**Authors:** Thomas Korn, Christoph Kleinschnitz, Tim Magnus, Sven G. Meuth, Ralf A. Linker, On behalf of the speakers at the 7th NEUROWIND e.V. scientific meeting

**Affiliations:** Department of Neurology, Klinikum rechts der Isar, Technische Universität München, Ismaninger Str. 22, 81675 München, Germany; Department of Neurology, University Clinic of Würzburg, Josef-Schneider-Str. 11, 97080 Würzburg, Germany; Department of Neurology, University Clinic Hamburg-Eppendorf, Martinistr. 52, 20246 Hamburg, Germany; Department of Neurology, University of Münster, Albert-Schweitzer Campus 1, 48149 Münster, Germany; Department of Neurology, Friedrich-Alexander-Universität Erlangen, Schwabachanlage 6, 91054 Erlangen, Germany

## Abstract

From October 30–November 1, 2015, the 7th NEUROWIND e.V. meeting was held in Motzen, Brandenburg, Germany. Seventy doctoral students and postdocs from over 25 different groups working in German and Swiss University Hospitals or Research Institutes attended the meeting to discuss their latest experiments and findings in the fields of neuroimmunology, neurodegeneration and neurovascular research. This meeting report summarizes the many diverse presentations and the new preclinical to clinical neurology research data that were shared by the participants at the meeting.

The meeting was regarded as a very well organized platform to support research of young investigators in Germany and all participants enjoyed the stimulating environment for lively in-depth discussions.

According to the major aim of the Association for the Advancement of Young Academics in Neurology (Verein zur Förderung des Wissenschaftlichen Nachwuchses in der Neurologie, NEUROWIND e.V.) to support younger researchers in Germany the 5th NEUROWIND YOUNG SCIENTIST AWARD for experimental neurology was awarded to Julia Bruttger (Group of Ari Waisman, Institute of Molecular Medicine, Johannes Gutenberg University Mainz, Germany) and Daniel von Bornstädt (Department of Neurology, Charité Berlin, Germany, Chair: Mathias Endres). Julia’s successful project was published in Immunity entitled “Genetic cell ablation reveals clusters of local self-renewing microglia in the mammalian central nervous system”. This outstanding paper deals with microglia repopulation in the adult CNS, relying on CNS-resident cells, independent from bone-marrow-derived precursors. Daniel’s work entitled “Supply–demand mismatch transients in susceptible peri-infarct hot zones explain the origins of spreading injury depolarizations” was published in Neuron. This outstanding work shows that somatosensory activation of peri-infarct cortex triggers peri-infarct depolarization when the activated cortex is within a critical range of ischemia. Both awards were endowed with 20,000 Euro sponsored by Merck Serono GmbH, Darmstadt, Germany (unrestricted educational grant).

This year’s keynote lecture was given by Joseph Classen, Head of the Department of Neurology at the University Hospital Leipzig. Dr. Classen highlighted the potential of repetitive transcranial magnetic stimulation or constant transcranial direct current stimulation in neuro-psychiatric diseases.

## Summary of the scientific presentations to the NEUROWIND e.V. meeting 2015

Lars Tönges (Bochum) reported on novel therapeutic targets for neuroprotection in Parkinson’s disease. Rho kinase (Rock) was identified as relevant mediator of neurodegeneration but also axonal regneration in the optic nerve crush model. This concept was transferred into the Parkinson’s disease model of MPTP mediated complex I inhibition and 6-OH dopamine lesion in the stereotactic striatal lesion model. Here, inhibition of Rock by Fasudil or application of AAV shRock2 vectors promoted recovery. Since Rock is expressed both in neuronal cells and in glial cells, the relevant cellular target of ROCK inhibition remains to be determined in the CNS.

Aiden Haghikia (Bochum) presented data on the modulation of the immune response by short and long chain fatty acids. While long chain fatty acids (e.g. laurate) promoted the generation of Th17 cells in the small intestinal lamina propria, short chain fatty acids enhanced the development of Foxp3 regulatory T cells (Tregs). Under physiologic conditions, the abundance of these fatty acid species was determined by certain taxa of the gut microbiome. Since dietary administration of these compounds aggravated or reduced EAE, respectively, this observation might bear some therapeutic potential, in particular because human T cells can be modulated accordingly by dietary intake of short chain fatty acids (e.g. propionate).

Kathrin Doppler (Würzburg) demonstrated that a subset of chronic inflammatory demyelinating polyneuropathy (CIDP) patients (about 5 %) harbored anti-contactin-1 antibodies against a protein expressed at the node of Ranvier. She was able to show that these antibodies (frequently of the subtype IgG4) were able to bind complement at the site of their binding on teased sciatic nerve preparations. Interestingly, the subset of anti-contactin-1 positive CIDP patients appeared to exhibit a distinct clinical phenotype with a relapsing remitting form of CIDP following an initial episode of “Guillain Barré like-syndrome”. Some patients displayed a tremor. In sural nerve biopsy, axonal loss was detected. Most CIDP patients with anti-contactin-1 serum antibodies responded well to rituxan.

Holger Haselmann (Jena) presented novel data on the functional role of anti-α-amino-3-hydroxy-5-methyl-4-isoxazolepropionic acid (AMPA) receptor antibodies on the AMPA receptor. Anti-AMPA receptor antibodies can occur as paraneoplastic phenomenon in association with tumors (64 %) and are able to cause limbic encephalitis. They bind the GluR2 subunit and impair the synaptic transmission and recovery after desensitization of the AMPA receptor in dissociated hippocampal cells. This was tested by single synapse iontophoretic glutamate patch clamp techniques analyzing evoked fast excitatory postsynaptic currents (eEPSC) in the presence or absence of anti-AMPA receptor antibodies. Together with the decrease of mEPSC amplitude and frequency in the presence of anti-GluR2 this was interpreted as loss of active receptors in the synapse.

Sarah Glumm (Münster) introduced a novel compound, WMS14–10, for the blockade of *N*-methyl-d-aspartic acid (NMDA) receptors. Similar to Ifenprodil, WMS14–10 binds to the 2B subunit of the NMDA receptor, but WMS is more selective. WMS14–10 was used in a prophylactic and therapeutic treatment regimen in experimental autoimmune encephalomyelitis (EAE) and was capable to significantly reduce the disease burden. Notably, WMS may directly act on microglia, which express GluN2B and down-regulate CD40, CD86 and MHCII upon exposure to WMS14–10.

Kai Diederich (Münster) shared his results on the treatment of mice with anti-leucine rich repeat and immunoglobin-like domain-containing protein (Lingo)-1 after stroke. The rationale for the use of anti-Lingo-1 in stroke is the observation that anti-Lingo-1 might improve the survival of neuronal and glial precursor cells that can be found in the border areas of a stroke lesion and thus promote regeneration. This idea was tested in pulse-chase experiments to label proliferating cells distinguishing cells that started proliferation before stroke (and would therefore represent the homeostatic rate of proliferating cells) and after stroke to visualize those cells that responded to the lesion. Anti-Lingo-1 treatment improved the survival of neuronal cells that started proliferation early post stroke. Notably, the administration of anti-Lingo-1 to mice after stroke was associated with an improved functional outcome of these mice in the tape removal test.

Gemma Llovera (Munich) reported on an alternative route of cell recruitment into an ischemic lesion. By using a photo-activatable green fluorescent protein mouse, she could show that immune cells that were activated by blue light irradiation with a probe in the choroid plexus were recruited to a distant ischemic lesion. Within the ischemic lesion, a number of chemokines including chemokine (C–C Motif) ligand (CCL) 2, 7, and 12 were found to be up-regulated and it is possible that in particular microglia that highly express CCL2 after stroke are important to attract immune cells including T cells from the ipsilateral choroid plexus. At this point, it is unclear how T cells recruited from the choroid plexus differ from T cells that are recruited through the blood brain barrier directly at the site of ischemia.

Nicolas Page (Geneva) presented his results on a novel mouse model of CD8 T cell driven auto-immunopathology in the central nervous system (CNS). In his model, the ODC-OVA mouse which expresses ovalbumin (Ova) in oligodendrocytes under the control of the MBP promoter was seeded with OT-1 cells and subsequently infected with either Lymphocytic choriomeningitis (LCMV)-OVA or Listeria monocytogenes (Lm)-OVA. After clearance of the infectious agent, LCMV-OVA infected but not Lm-OVA infected mice developed CNS pathology while OT-1 cells were identified in the CNS in both cases. By differential gene expression profiling of these OT-1 cells, highly interesting novel genes were identified that are now validated for their potential to drive encephalitogenic properties of CNS infiltrating CTLs.

Nikola Wilck (Berlin) reported on his observations regarding dietary short chain fatty acids on the cardiovascular system. He proposed the hypothesis that propionate ameliorated Angiotensin II driven cardiac damage and he provided evidence that dietary treatment with propionate had an immune mediated effect on the heart muscle by altering the balance of pro- vs anti-inflammatory T cell subsets. It remains to be determined which molecular pathway (including G-protein coupled receptor 41, 43, 109A or modulation of histone deacetylase) is used by propionate to exert its effects on immune cells.

Kavi Devraj (Frankfurt) introduced his in vitro system to measure effects of S1P modulators on endothelial cells in the context of the blood brain barrier. In mouse brain endothelial cells (MBEC), he could show that S1P1 blockers have a biphasic effect on the trans-endothelial resistance: An early tightening and a late opening effect. The late effect of S1P1 modulators appeared to be mediated by VE-cadherin.

Anna Hammer (Erlangen) introduced the role of the receptor Mas in neuroinflammation. Mas is a G-protein coupled receptor of angiotensin (Ang)1–7 which can be generated from Ang II by the action of Ang converting enzyme 2. Ang1–7 binds Mas and induces vasodilatation. Since intervention in the renin/angiotensin axis has been shown to be beneficial in neuroinflammation, Hammer and colleagues decided to test the role of Mas in EAE. Mas−/− mice develop a more severe EAE as compared to littermate controls. Since Mas is expressed on macrophages, the authors focused on the role of Ang1–7 in macrophage differentiation and—based on expression profiling of wild type vs Mas knock-out macrophages—proposed the hypothesis that Mas mediated effects rather promote a M2 differentiation of macrophages.

Stefanie Kürten (Würzburg) showed her study on the role of Carcinoembryonic antigen-related cell adhesion molecule (CEACAM) 1 on B cells during chronic autoimmune neuroinflammation. In a model of EAE induced with an MBP-PLP fusion protein (MP4), tertiary lymphoid aggregates emerge in the meningeal space and it has been proposed that the presence of these aggregates is associated with a progressive disease course in this model. Since CEACAM 1 was identified to be expressed on B cells, a depleting anti-CEACAM 1 antibody was tested to interfere with the clinical course in this model. In fact, anti-CEACAM 1 treatment reduced the number of newly formed tertiary lymphoid aggregates in the cerebellar parenchyma without affecting the peripheral B and T cell responses. Chronic disease symptoms were reduced by anti-CEACAM 1 treatment. Notably, MS patients harbor an increased fraction of CEACAM 1+ B cells in their circulating pool of naive B cells, memory B cells and B1 cells. Finally, in histologic specimens of SPMS patients CEACAM 1+ cells can be identified within meningeal aggregates of immune cells suggesting that anti-CEACAM 1 treatment might represent a means to interfere with the formation of B cell aggregates in MS patients as well.

Sabine Pfeifenbring (Göttingen) investigated axonal degeneration in children with MS to figure out quantitative differences in axonal damage between pediatric and adult MS patients which might explain the clinical specificities of pediatric MS. Early MS lesions from pediatric patients were characterized according to demyelinating activity and presence of remyelination. Axonal damage was assessed using Bielschowsky’s silver impregnation and immunohistochemistry for amyloid precursor protein (APP). Biopsy and autopsy tissue from 19 children with MS were compared with 12 adult MS patients. Acute axonal damage was more extensive in children compared to adults. The highest extent of acute axonal damage was detectable in early active lesions of prepubertal patients, while this group of patients also revealed the densest infiltration of macrophages/microglia within the lesions. The numbers of T lymphocytes did not significantly differ between pediatric and adult MS patients. Furthermore, a higher EDSS at attack leading to biopsy or autopsy was associated with a higher extent of acute axonal damage. Hence, the extent of acute axonal damage seems to be one of the factors responsible for the often observed severe disease onset in pediatric MS.

Claudia Dames (Berlin) reported on her work on post stroke immunosuppression. After stroke, the parasympathetic tone is increased. The relevance of this parasympathetic hyperactivity for immunosuppression was tested in a model of suboptimal bacterial pneumonia in post stroke mice. Vagotomy before stroke restored peripheral immunocompetence, while opposite effects were observed in nicotinic acetylcholine receptor α7 knock-out mice. Interstitial SiglecF+F4/80+ (CD11c−) cells in the lung were significantly reduced during several days post stroke. Stroke mice harbored increased levels of albumin in the bronchoalveolar lavage as a consequence of loss of barrier function. In addition, stroke mice had decreased muco-ciliary clearance and were—perhaps also due to reduced numbers of interstitial macrophages—incapable to clear a given bacterial burden. Sympathetic stimuli (nicotine) partly reversed post stroke immunosuppression.

Michael Bieber (Würzburg) presented data on stroke-induced cardiac failure. Immediately after stroke, not only the parasympathetic but also the sympathetic tone is increased resulting in increased levels of catecholamines in the peripheral circulation. In humans, it has been reported that stroke affects the function of the heart. In a preclinical model of post-stroke hypersympathetic tone (at 8 weeks after stroke), myocardial fractional shortening and ejection fraction were decreased whereas brain natriuretic peptide expression as well as matrix metallopeptidase 9 and TNF-α were increased in myocardial tissue. Interestingly, the cardiac phenotype post stroke was reversed by beta blockers. In parallel to ongoing mechanistic studies in this pre-clinical model, a large observational clinical trial was initiated to monitor heart affection after stroke.

Sarah Brockmann (Ulm) gave an overview on new genes linked to amyotrophic lateral sclerosis (ALS). While the fraction of ALS patients that can be tracked down to harbor genetic variants of either SOD or C9orf72 has increased over the past years, the discovery of novel mutations in ALS patients by exome sequencing will likely increase the fraction of genetically determined ALS even more in the future. CHCHD10 is a protein that is targeted to the crista junction in the membranes of mitochondria. It has been speculated that pathogenetic mutations of CHCHD10 interfered with the respiratory chain. However, more work is required as to which mutations of CHCHD10 are really pathogenic and what their downstream targets are.

Fabian Szepanowski (Düsseldorf) shared his data on the impact of fingolimod on peripheral nerve regeneration. Sphingosine 1 phosphate receptor modulation was investigated in the sciatic nerve crush model in wild-type vs recombination-activating gene 1 knock-out (neither B nor T cells) vs forkhead box N1 knock-out (no T cells) mice. Fingolimod treatment was started 2 days before crush until 2 weeks after crush, and nerve conduction velocity was measured. It increased cyclic adenosine monophosphate levels in crushed nerves independently of the presence of T or B cells. Moreover, fingolimod inhibited lysophosphatidic acid synthesis and thereby improved remyelination with increased g-ratios in peripheral myelinated axons. Given these positive results it remains a speculation that the failure of fingolimod to exert neuroprotective properties in primary progressive multiple sclerosis (INFORMS trial) might be due to the fact that the concentrations of fingolimod that were reached within the CNS were too low to be operational.

Ann-Kathrin Pulm (Düsseldorf–Münster) talked about her analysis of the role of endogenous interferon (IFN)β during EAE. Using a reporter mouse for IFNβ (IRES-YFP), it was shown that microglia were a relevant source of IFNβ at day 17 (peak of disease) in EAE. These activated microglia were identified as orchestrators of INFβ-induced clearance of myelin debris.

Mathias Gelderblom (Hamburg) presented his analysis of the role of dendritic cells (DC) in stroke. Dendritic cells are recruited early after stroke. Their infiltration peaked at day 3 and decreased by day 7 after stroke. The population composition of DCs changed during post stroke infiltration. While CD103−Sirp1a+ DCs dominate first and are then replaced by CD103+CD11b+XCR1+ DCs. Using the CD11c− diphteria toxin receptor mouse (CD11c.DOG) model, DCs were depleted before stroke, which led to smaller lesions. Notably, DC depleted mice had post stroke infiltrates with less gamma delta (γδ) T17 cells and less neutrophils suggesting that DCs might activate γδT17 cells to license neutrophil infiltration post stroke. In fact, DCs are the main producers of IL-23 in the post stroke infiltrate and IL-23R knock-out mice recapitulated the phenotype of DC depleted mice in the stroke model.

Claudia Lange (Greifswald) gave an overview over the effects of stress hormones on circulating immune cells after stroke. Granulocytes collected from patients immediately after an ischemic stroke showed an impaired oxidative burst after N-formyl-methionyl-leucyl-phenylalanine stimulation and decreased necosis (area covered by DNA nets) after phorbol 12-myristate 13-acetate stimulation. These effects can be mimicked by incubation of granulocytes from healthy donors with noradrenaline while acetylcholine increases effector functions of granulocytes. Similarly, the cytotoxic activity of NK cells was reduced in response to stress hormones.

Karin Loser (Münster) reported on therapeutic approaches in neuroinflammation that exploit the beneficial effect of UV light in EAE and MS. NDP-MSH is a more stable derivative of α-MSH (alpha-melanocyte stimulating hormone) that also is a more potent agonist to the melanocortin1 receptor (MC1R). NDP-MSH improved EAE be pleiotropic mechanisms. First, it expanded Foxp3+ Tregs. Second, it increased the laminin5 expression along the blood brain barrier to a continuous pattern and third, by up-regulating nuclear receptor subfamily 4 in neurons, NDP-MSH protected neurons from excitotoxicity. Interestingly, the immune mediated effects of NDP-MSH were recapitulated in the OSE mouse model of spontaneous EAE.

Katharina Ochs (Heidelberg) presented a study on the co-incidence of MS and glial tumors. 23 patients with both diseases were identified. In 80 % of cases, MS preceded the onset of the tumor. All patients had a relapsing MS course and the tumor entities were not biased towards a particular type of glioma (based on methylation epigenetics). The course of MS was likely not altered by the co-morbidity of the tumor although radiotherapy might be associated with increased relapse activity.

Christoph Harms (Berlin) presented novel data on the presence of an isoform of phosphatase and tensin homolog (PTEN; PTEN long) in neurons. Classic PTEN interferes with the phosphatidylinositide 3-kinase (PI3K) pathway by inhibiting the conversion of phosphatidylinositol-bisphosphate to phosphatidylinositol trisphosphate. The new isoform PTEN long exists in neurons and also inhibits the PI3K/Akt pathway. Gain of function by transduction of PTEN classic or PTEN long into PTEN deficient neurons revealed that both isoforms appeared to be redundant, i.e. a loss of Akt phosphorylation was detected in both cases (WB on pAkt). In vitro, in the oxygen and glucose deprivation model, PTEN deficient neurons were rescued by both transduction of PTEN classic and PTEN long, again suggesting that both isoforms are redundant. Further experiments have to address whether PTEN long has a distinct function in neurons.

In conclusion, this year’s NEUROWIND meeting once again managed to be an exciting platform for bringing together highly motivated young scientists in experimental neurology to discuss scientific progress in the fields of neuroimmunology, vascular biology, and neurodegeneration. All participants and the organizers are now looking forward to the upcoming NEUROWIND e.V. meeting in 2016.

